# Population Health and Health Services: Old Challenges and New Realities in the COVID-19 Era

**DOI:** 10.3390/ijerph18041658

**Published:** 2021-02-09

**Authors:** Antonio Sarría-Santamera, Alua Yeskendir, Tilektes Maulenkul, Binur Orazumbekova, Abduzhappar Gaipov, Iñaki Imaz-Iglesia, Lorena Pinilla-Navas, Teresa Moreno-Casbas, Teresa Corral

**Affiliations:** 1 Department of Medicine, Nazarbayev University School of Medicine, Nur-Sultan 02000, Kazakhstan; alua.yeskendir@nu.edu.kz (A.Y.); tilektes.maulenkul@nu.edu.kz (T.M.); binur.orazumbekova@nu.edu.kz (B.O.); abduzhappar.gaipov@nu.edu.kz (A.G.); 2 Spanish Network in Health Services Research and Chronic Diseases (REDISSEC), 28029 Madrid, Spain; imaz@isciii.es (I.I.-I.); Lorena.pinilla892@gmail.com (L.P.-N.); 3 Institute of Health Carlos III (ISCIII), 28029 Madrid, Spain; mmoreno@isciii.es (T.M.-C.); tcorral@isciii.es (T.C.); 4 Center for Biomedical Research in Frailty and Health Aging (CIBERFES), 28029 Madrid, Spain

**Keywords:** delivery of health care 2, health service, preventive 3, health promotion 4, health policy

## Abstract

(1) Background: Health services that were already under pressure before the COVID-19 pandemic to maximize its impact on population health, have not only the imperative to remain resilient and sustainable and be prepared for future waves of the virus, but to take advantage of the learnings from the pandemic to re-configure and support the greatest possible improvements. (2) Methods: A review of articles published by the Special Issue on Population Health and Health Services to identify main drivers for improving the contribution of health services on population health is conducted. (3) Health services have to focus not just on providing the best care to health problems but to improve its focus on health promotion and disease prevention. (4) Conclusions: Implementing innovative but complex solutions to address the problems can hardly be achieved without a multilevel and multisectoral deliberative debate. The CHRODIS PLUS policy dialog method can help standardize policy-making procedures and improve network governance, offering a proven method to strengthen the impact of health services on population health, which in the post-COVID era is more necessary than ever.

COVID-19 is the biggest challenge that our societies have faced in living memory. Across the world, the COVID-19 pandemic is having a tremendous impact on our societies and health care systems. Even affluent countries are facing critical challenges, with governments having to rapidly pivot resources and bring in extra protections for groups at risk while seeking care for COVID-19, with mixed success. Health systems that were already under pressure before the pandemic, have not only the imperative to remain resilient and sustainable while continuing to be prepared for future waves of the virus but to take advantage of the learnings from the pandemic to re-configure and support the greatest possible improvements, well beyond this crisis.

We may define population health as the health outcomes of a group of individuals, including the distribution of such outcomes within the group [1]. Populations may be defined as geographic regions, like nations or communities, but we can define them to be other groups, such as employees, ethnic groups, disabled persons, or prisoners. Population health research aims to understand health, diseases, and their determinants, developing appropriate methodological approaches and analyzing the current issues affecting human health from different perspectives.

Population health is about creating a collective sense of responsibility across many organizations and individuals. It brings together a diverse range of professionals and disciplines, with a common aim of understanding, safeguarding, and improving the health of populations and individuals through education, cooperation, and research. The COVID-19 pandemic has highlighted the importance of a population approach to tackle unexpected health threats. Four pillars have been proposed for population health: the broader determinants of health; health behaviors and lifestyles; the places and communities we live in; and the health services [2].

This Special Issue of the IJERPH has been dedicated to exploring different angles and perspectives of the relationship between population health and one of those pillars: health services. Papers published in this Special Issue offer not just a rich sample of the wide relationship between the health of the populations and health services, but take-home lessons for improving the impact of health services on population health.

Health services, organizations, people, and actions whose primary intent is to promote, restore, or maintain health [3], have to play a critical role in improving population health with a consistent orientation towards health promotion and disease prevention [4]. Core elements of the population health approach include a focus on improving health and wellbeing rather than curing illnesses, understanding needs and solutions through a community perspective, with a life-cycle perspective, and a focus on vulnerable groups, addressing the social determinants of health and inter-sectoral partnerships and promoting healthier lifestyles and behaviors. Van Dale et al. show in their paper a series of key factors to strengthen health services engagement in community partnerships: inter-connecting with existing policies, defining a shared vision, creating an effective mix of different partners, encouraging effective leadership, keeping collaboration partners engaged, using a planned systematic approach, and ensuring sufficient resources [5].

As well as preventing chronic conditions, health services contribute to population health through the appropriate management of problems once they are diagnosed, aiming towards secondary and tertiary prevention. In our societies, chronic diseases represent a major burden for patients, their families, health care systems, and the society at large. As Wilczyński et al. reflect, preventive interventions have to address from a life-cycle perspective the entire range of determinants associated with those problems [6].

Evaluation of chronic disease programs and interventions is critical [7]. Improving effectiveness and patient outcomes when treating complex conditions, as Carrasco-Peña et al. identify, is strongly influenced by adherence to quality standards and guidelines [8]. In chronic disease management, it is fundamental to develop appropriate methodological approaches to identify patient and health care system structures and processes of care related to outcomes [9]. Nakamura et al. capture in their paper the critical role of new technologies that are going to maximize effective and efficient care [10], but also the existing differences in how those systems are used by different population groups.

A growing concern is the continuos grow in the complexity of long-term contions, as the paper by Ioakeim-Skoufa indicates [11]. Rodríguez-Blázquez et al. reports on the implementation of a multimorbidity care model in several countries, and regardless of the significant diversity in organizational aspects of the different settings where this model was implemented, there was a consistent improvement in the quality of care indicating the need to integrate an orientation towards multimorbidity in our health care systems [12].

The paper by Sarría-Santamera et al. reflects on the need to better identify patients’ sub-populations for diseases, like diabetes, with such a significant population impact, to target as many as possible treatments, linking patient phenotypic characteristics with treatments whose mechanism of action may be better fitted to their specific metabolic disturbances. The findings of Sanfillippo et al. are also relevant, reflecting a combination of variability in clinical practice in the management of patients with chest pain, and increasing use of coronary procedures in those patients even with normal troponin levels, showing that still further investigation is required to determine the risk profile, outcomes, and cost-effectiveness on managing these patients [13]. Wei and Zhang and Lv et al. discuss in their respective papers how different factors, at the individual level, like aging, presence of chronic diseases, education, residence, income, and self-care ability, as well as other factors, related to structural and social components, influence the utilization of health systems [14,15].

Health systems worldwide face increasing challenges from the rising costs of care, a growing number of elderly living with complex multimorbid problems, and the recognition of a failure to implement effective health promotion and disease prevention interventions. Health services have to adopt technological innovations [16] while controlling the overuse of health services [17], as well as advance the integrating health and non-health services (and resources) to coordinate actions among health care and public health services, social and community organizations [18].

Digitalization of health services has reached a new level during the pandemic. Digital technologies have become irreplaceable tools in pandemic response, management, and control: real-time monitoring systems, migration maps, data dashboards, real-time data collection devices, and artificial intelligence (AI) have been integrated into different steps of pandemic control, including surveillance, contact tracing, quarantine, testing, and clinical management [19]. Internet of Things, Big Data, Machine Learning, and AI are changing the delivery of health services. AI could be very effective in providing faster decision-making in diagnosis, treatment, and day-to-day monitoring of the COVID-19 cases and suspects, which can be especially valuable when health care professionals and health care systems experience extremely high workloads [20].

Digital technologies and data-driven decision-making, predictive health care based on big data analysis, telemedicine, wearable medical devices, and smartphone applications, may transform health services delivery by improving accessibility, making them less prone to human errors and more cost-effective. Remote virtual health care can become a game-changing tool in preventive medicine and management of chronic diseases.

The absence of effective treatment also prompted researchers to quickly search for therapeutics against COVID-19. AI algorithms based on big data analysis showed promising results in the fast identification of potential therapeutics with anti-viral properties and candidate vaccines [21]. However, the COVID-19 crisis has also revealed the unpreparedness not just of governments and health services, but also of biopharma industries: pharmaceutical companies invest more in medications against oncology, immunology, and cardiovascular diseases compared to infectious disease medications [22]. The current pandemic showed the danger and price of not having available vaccines and effective anti-infectious drugs and consequently the need to increase investments in infectious disease programs [23]. Institutions like the European Union have reacted and created initiatives, like the Global Research Collaboration for Infectious Disease Preparedness (GloPID-R), to address this problem [24].

Another important lesson from the COVID-19 pandemic are the problems of governments and local pharmaceutical companies with the supply of population with essential drugs, medical devices, and personal protective equipment. China and India are among the biggest producers of active pharmaceuticals in the world, and closure of their borders during the pandemic slowed down the production of certain medicines and increased drug prices [25]. Supply disruptions and medicine shortages during the pandemic may prompt governments to more concentrate on the production of their own essential therapeutics and medical devices to avoid future crisis.

Mental health is another critical area hardly hit by COVID-19. Social isolation, remote education and working, loss of income, limited physical activity, increased access to food and drinks, financial and emotional insecurity, and absence of social support can cause a variety of psychological problems including but not limited to distress, insomnia, anxiety, depression, eating disorders, and exacerbation of existing chronic conditions [26], worsening of psychiatric symptoms of individuals with preexisting mental disorders, symptoms of anxiety, depression, both among the general public and health care workers [27]. Those problems were persistent even after the quarantine and led to long-term behavioral changes [28].

The COVID-19 outbreak has reduced the possibility of traditional face-to-face care; thus, some countries have already introduced online mental health services and remote psychological interventions [29,30]. Shifting from the traditional way of mental health services delivery can cause some issues concerning confidentiality, data security, internet access, and ability to use technologies; even more, some people can struggle to interact during online sessions and for some individuals with lower digital literacy or specific health conditions it could be impossible to receive mental health services remotely [23]. Although, online mental health care has already shown some positive outcomes [27,31], remote therapy could not work for everyone and demands a more personal approach from the physician’s site. Long-lasting lockdowns and social isolation have revealed new potential of preventive mental health strategies in the forms of family and community support, and self-care [23]. It will be highly valuable making sure to establishing intersectoral links between different health services.

Implementing innovative but complex solutions to address the problems mentioned above is not simple. Sienkiewicz et al. describe how the CHRODIS PLUS policy dialogs have proved an effective mechanism to provoke deliberative discussion on a wide range of health policy topics in different settings, stimulating thought and concrete actions about priorities and rationales [32]. The suggested method helped to keep stakeholders engaged, raise their awareness of needs, challenges, and opportunities, setting concrete goals and objectives for a wide variety of health policy issues. The complex challenges that our health services face can hardly be achieved without a multilevel and multisectoral deliberative debate. The CHRODIS PLUS methodology can help standardize policy-making procedures and improve network governance through greater dialogue and civic engagement, offering a proven method to strengthen the impact of health services on population health, which in the post-COVID era is more necessary than ever.
